# Video-Delivered Family Therapy for Home Visited Young Mothers With Perinatal Depressive Symptoms: Quasi-Experimental Implementation-Effectiveness Hybrid Trial

**DOI:** 10.2196/11513

**Published:** 2018-12-10

**Authors:** Fallon Cluxton-Keller, Melony Williams, Jennifer Buteau, Craig L Donnelly, Patricia Stolte, Maggie Monroe-Cassel, Martha L Bruce

**Affiliations:** 1 Department of Psychiatry Geisel School of Medicine at Dartmouth College Lebanon, NH United States; 2 FRC Claremont, NH United States; 3 FRC Gorham, NH United States

**Keywords:** videoconferencing, family therapy, depression

## Abstract

**Background:**

The Federal Maternal, Infant, and Early Childhood Home Visiting Program is a national child abuse prevention strategy that serves families at risk for child maltreatment throughout the United States. Significant portions of the clients are young mothers who screen positive for clinically significant perinatal depressive symptoms and experience relational discord that worsens their symptoms. Although home visitors refer those who screen positive for depression to community-based treatment, they infrequently obtain treatment because of multiple barriers. These barriers are compounded for home visited families in rural areas.

**Objective:**

This pilot study aimed to explore the feasibility, acceptability, and effectiveness of a video-delivered family therapy intervention on reducing maternal depressive symptoms and improving family functioning and emotion regulation.

**Methods:**

A total of 13 home visited families received the video-delivered family therapy intervention. This study included a historical comparison group of mothers (N=13) who were previously enrolled in home visiting and screened positive for clinically significant perinatal depressive symptoms but refused treatment. A licensed marriage and family therapist delivered the family therapy intervention using Health Insurance Portability and Accountability Act–compliant videoconferencing technology on a computer from an office. Families participated in sessions in their homes using cell phones, tablets, and computers equipped with microphones and video cameras. Outcomes were measured following the final therapy session (post intervention) and 2 months later (follow-up). Depressive symptom scores of mothers who received the video-delivered family therapy intervention were compared with those of mothers in the historical comparison group over a 6-month period. Univariate statistics and correlations were calculated to assess measures of feasibility. Percentages and qualitative thematic analysis were used to assess acceptability. Wilcoxon signed-rank tests were used to assess changes in maternal and family outcomes.

**Results:**

No families dropped out of the study. All families reported that the technology was convenient and easy to use. All families reported high satisfaction with the video-delivered intervention. Nearly all families reported that they preferred video-delivered family therapy instead of clinic-based therapy. Therapeutic alliance was strong. Mothers demonstrated a statistically significant reduction in depressive symptoms (*P*=.001). When compared with mothers in the historical comparison group, those in the family therapy intervention showed a significant reduction in depressive symptoms (*P*=.001). Families demonstrated statistically significant improvements in family functioning (*P*=.02) and cognitive reappraisal (*P*=.004).

**Conclusions:**

This pilot study yielded preliminary findings that support the feasibility, acceptability, and effectiveness of the video-delivered family therapy intervention for underserved home visited families in rural areas. Our findings are very promising, but more research is needed to ultimately influence mental health practices and policies that pertain to video-delivered mental health interventions in unsupervised settings (eg, homes).

## Introduction

### Background

The Federal Maternal, Infant, and Early Childhood Home Visiting (MIECHV) Program serves over 100,000 vulnerable families at risk for child abuse throughout the United States and aims to improve several outcomes, including maternal mental health [1]. Over half of the families served include young mothers (pregnant and postdelivery) under the age of 25 years [2]. Depressed young mothers make up a significant portion of home visited clients. Between 34% and 60% of home visited young mothers screen positive for clinically significant perinatal depressive symptoms [3-7]. Home visited mothers infrequently access treatment because of barriers (eg, no child care, lack of transportation, geographical distance, and stigma) [8-10]. These logistical barriers are compounded for mothers in rural regions.

Home visitors refer depressed mothers for treatment but they infrequently obtain it or complete it [7-12]. Between 8% and 32% of depressed home visited mothers report receipt of some mental health treatment [3,6,7,12-14]. One study showed that about 19% of home visited mothers recover from depression as a result of the illness’s natural course, reduced stressors, or mental health treatment in the community [3]. Untreated maternal depression leads to poor maternal and child outcomes [15,16]. Furthermore, studies have shown that home visiting outcomes are very poor for mothers with severe depressive symptoms and high levels of discomfort with trust. One study showed that home visiting did not impact cognitive development or behavior in children of mothers with both severe depressive symptoms and high levels of discomfort with trust [17]. Another study showed that in depressed mothers with both low relationship anxiety and high discomfort with trust, home visiting increased the likelihood of substantiated child maltreatment [18]. Untreated maternal depression is associated with child abuse and neglect [19,20].

Integrating mental health services in home visiting has been shown to reduce logistical barriers experienced by some families [8-10]. There are currently 4 individual-level psychosocial interventions that target maternal depression as a primary outcome [21,22,24]. One intervention has been delivered in a group format [23] and requires mothers to travel to the location of the group. The other 3 interventions require providers to travel to mothers’ homes [21,22,24]. Feasibility and sustainability problems exist with these current interventions [21-24] in rural areas because of even greater provider/maternal travel time that nearly triples the cost of the intervention. MIECHV serves families in 22% of all rural US counties [2], and with enrollment expected to increase in the coming years, the delivery method of these existing models [21-24] is likely not feasible or sustainable in rural areas.

Furthermore, these existing interventions do not include family members in the mother’s treatment to address family dynamics that result in family conflict. Family conflict is a risk factor for and a consequence of perinatal depression [25-32]. Family conflict (nonviolent) precipitates clinically significant perinatal depressive symptoms in adolescent and young adult mothers [25,28-31,33]. Family conflict (eg, frequent arguments and criticism) worsens perinatal depressive symptoms in young mothers, and if conflict is untreated, their depressive symptoms often persist [26,30,31,33-37].

Technology permits convenient access to treatment for mothers and their families in rural areas. Given that 60% of low-income families and nearly 68% of those in rural areas own handheld devices with internet access in the United States [38], technology bypasses logistical barriers to increase treatment access and eliminates the clinician’s travel time to family homes. The use of Health Insurance Portability and Accountability Act (HIPAA)–compliant video-based communication technology is not novel in rural areas and ample evidence supports its use (eg, [39,40]). Studies have been conducted on video-delivered family interventions for some populations [41-44], but none have targeted perinatal depressive symptoms in home visited mothers [45].

### Aims of This Study

This 1-year pilot study had the following 2 aims: (1) to explore the feasibility and acceptability of the video-delivered family therapy intervention among home visited families and (2) to explore preliminary impacts of the video-delivered family therapy intervention on maternal depressive symptoms, family functioning, and emotion regulation from baseline to 2 months after the final family therapy session (follow-up). We hypothesize that mothers will show a clinically meaningful reduction in depressive symptoms at the 2-month follow-up [46]. When compared with depressed mothers who were previously enrolled in home visiting but refused treatment, those who received the video-delivered family therapy intervention will show preliminary evidence of a greater reduction in depression [46]. Finally, we hypothesize that families will show clinically meaningful improvement in family functioning and emotion regulation at the 2-month follow-up [46].

As described in our published study protocol [46], our research trial originally included maternal attitudes toward parenting practices as an outcome. Unfortunately, the measure (Adult-Adolescent Parenting Inventory, second edition) that was selected for this outcome was found to be unreliable with the home visited mothers that participated in our pilot study. The principal investigator (PI; first author) received reports from research staff that mothers did not understand the items, and the Cronbach alphas for the different subscales of this measure reflected the mothers’ poor understanding of the items. For these reasons, this outcome could not be tested and was eliminated from this study. Our research trial included an additional aim to explore the feasibility and acceptability of integrating the video-delivered family therapy intervention into home visiting [46]. The findings for this additional aim will be reported in a subsequent manuscript.

## Methods

### Study Design

This study is a quasi-experimental, implementation-effectiveness hybrid trial. Although participants were not randomized, our study included a historical comparison group of mothers (n=13) who were previously enrolled in the home visiting programs at the 2 MIECHV agencies. The mothers in the historical comparison group were matched on baseline depression scores, age, and number of children. This study is registered at ClinicalTrials.gov (ID: NCT03282448).

### Participants

Families were recruited from 2 MIECHV agencies in New England. These 2 agencies serve families in 3 rural counties in New England. Home visitors presented an institutional review board (IRB)–approved study information sheet to eligible families in their caseloads that included a brief overview of the study goals, intervention, study measures, potential benefits, and risks. Home visitors provided each interested family’s contact information to research assistants, who scheduled a meeting with each family to review the IRB consent forms, and obtained written informed consent from all willing participants. During the consent process, families were informed that study participation was voluntary and they could receive home visiting regardless of whether or not they decided to participate in the study.

Of the 28 potentially eligible families, 13 were excluded from the study because of the severity of psychiatric illnesses (eg, pervasive developmental disorder and bipolar disorder), maternal age that exceeded the age cut-off, domestic violence, and recent completion of standard dialectical behavior therapy (DBT) treatment. Overall, 2 families were excluded before the first therapy session because the home visitor was unable to locate them after they signed the consent form. Our final sample size for the intervention group was 13 families.

Textbox 1 includes the family inclusion criteria and Textbox 2 includes the family exclusion criteria.

**Family inclusion criteria.**

**Inclusion criteria**

- Home visited mothers, ages 13-25, in the first trimester of pregnancy through 18 months postpartum;
- Mothers with *Edinburgh Postnatal Depression Scale* (*EPDS*) [47] scores of ≥8;
- At least one of the mother’s adult family members (“Family member” is defined as one who is biologically related to the mother or a significant close other with whom she is not biologically related) must be available to participate in 8 of the 10 sessions;
- Mothers and family members must be fluent in English;
- Families with consistent internet access (i.e., subscribe to an internet service provider and do not experience weekly disruptions in service); and
- Family owns cell phone, tablet or computer equipped with a camera and microphone.

**Family exclusion criteria.**

**Exclusion criteria**

- Current substance abuse in mothers;
- Current domestic violence;
- Mothers who report or exhibit current suicidal ideation, self-injurious behavior, and psychotic symptoms during a home visit or in a therapy session;
- Mothers experiencing a severe Major Depressive Episode;
- Mothers who report homicidal ideation to the home visitor or study therapist;
- Mothers with pervasive developmental disorders;
- Mothers with a diagnosis of Post-Traumatic Stress Disorder;
- Current family therapy;
- Current individual therapy for mothers or recent completion of Dialectical Behavior Therapy; and/or
- Child Protective Services (CPS) involvement as reported by the home visitor.

**Table 1:** Study measures by aim, construct, respondent, and time point.

Constructs		Measure	Respondent		Time point		
			Mother	Family	O_1_	O_2_	O_3_
**Feasibility**							
	Quality of the therapeutic alliance	Working Alliance Inventories-Short Forms therapist and client versions^a^ [50,51]	X	X		X	X
	Retention	Percent of families who complete treatment	X	X			X
**Acceptability**							
	Family	Percent of completed homework assignments	X	X		X	
	Responsiveness	Satisfaction Questionnaire^b^	X	X			X
**Effectiveness**							
	Depression	BDI-II^c^ [52]	X		X	X	X
	Family functioning	Protective Factors Survey, Family Functioning/ Resiliency subscale [53]	X	X	X		X
	Emotion regulation	Emotion Regulation Questionnaire [54]	X	X	X		X

^a^Monitoring measure, also completed by therapist, at the end of therapy session 6.

^b^Developed by research team.

^c^Beck Depression Inventory-second edition; monitoring measures completed at the end of therapy sessions 4 and 8.

This study used standard home visiting screening procedures for depression, substance abuse, and domestic violence to identify eligible families for participation. Home visitors are expected to routinely screen mothers for depression and provide mental health referrals to those who screen positive for depression on the Edinburgh Postnatal Depression Scale (EPDS). Home visitors are also expected to routinely screen mothers for substance abuse (eg, cut-annoyed-guilty-eye, CAGE [48]) and domestic violence (Relationship Assessment Scale [49]). Home visitors used the mothers’ most recent scores on these measures to determine if they were eligible for study participation.

### Data Collection

The study measures and time points that align with each aim are presented in Table 1. Study measures were completed at 3 time points: family baseline (O_1_); treatment phase (O_2_); and post intervention (immediately after the final therapy session) and 2 months later (follow-up; O_3_). Family baseline measures were completed in O_1_ within 3 weeks before the first therapy session. Demographic information was collected on mothers, infants (age only), and family members. Monitoring measures were completed in O_2_. Postintervention measures and the 2-month follow-up were completed in O_3_.

### Feasibility and Acceptability Measures

The Working Alliance Inventories-Short Forms (therapist and client versions) were used to measure quality of the therapeutic alliance. The developer recommends for therapists and clients to complete this measure beginning after the third, 60-min, therapy session [50,51]. As each family therapy session lasted only 30 min, the therapist and families completed it after the sixth family therapy session instead and also completed it after the final family therapy session, post intervention. The measure includes 3 subscales to evaluate the strength of the therapeutic alliance: goals, bond, and task [50,51]. Each subscale includes response choices that range from seldom to always, but the numeric order is reversed for some of the items [50,51]. Two of the subscales demonstrated good reliability for families at the session 6 time point (goals, Cronbach alpha=.92; bond, Cronbach alpha=.85). The third subscale did not demonstrate good reliability at the session 6 time point (task, Cronbach alpha=.43) for families but demonstrated good reliability post intervention (task, Cronbach alpha=.72). Family retention was measured by calculating the percentage of families who completed treatment.

The PI and coinvestigators developed the Satisfaction Questionnaire for this study. It includes 7 quantitative items that include 6 multiple-choice questions, with response choices that range from 1 (strongly agree) to 4 (strongly disagree), and the seventh question is rated as true or false. In total, 5 of the items assess overall satisfaction with the therapy and satisfaction with each of the 4 skills modules. The last 2 items are on the usefulness of the HIPAA-compliant video-based communication technology. The Satisfaction Questionnaire also includes 3 open-ended questions about changes families would like to see to the video-delivered family therapy intervention, the helpfulness of the skills, and a section for general comments. The Satisfaction Questionnaire was administered to families post intervention. All the quantitative items on it demonstrated good reliability (Cronbach alpha=.85) post intervention. Completion of homework assignments was based on the family report to the therapist. The percentages of families who completed homework assignments for each of the 4 skills modules were calculated.

### Outcome Measures

The Beck Depression Inventory-second edition (BDI-II) [52] was used to measure maternal depressive symptoms at baseline, post intervention, and at the 2-month follow-up. Maternal depressive symptoms were also monitored after the fourth and eighth therapy sessions using the BDI-II. This 21-item measure demonstrated good reliability in this study at baseline (Cronbach alpha=.88). The Protective Factors Survey-Family Functioning/ Resiliency subscale [53] was used to measure family functioning at baseline, post intervention, and at the 2-month follow-up in mothers and their family members. This 5-item measure includes items on verbal communication, collaborative problem solving, cohesion, and family conflict. Response choices range from 1 (never) to 7 (always), with higher scores indicating better functioning [53]. It demonstrated good reliability in this study at baseline (Cronbach alpha=.90). The Emotion Regulation Questionnaire [54] was used to measure emotion regulation, and it includes 2 subscales: cognitive reappraisal and expressive suppression. This 10-item measure includes response choices ranging from 1 (strongly disagree) to 7 (strongly agree), with higher scores indicating greater use of the emotion regulation strategy [54]. Emotion regulation was measured in mothers and their family members at baseline, post intervention, and at the 2-month follow-up. The cognitive reappraisal subscale demonstrated good reliability at baseline (Cronbach alpha=.81). The expressive suppression subscale demonstrated adequate reliability at baseline (Cronbach alpha=.68).

The EPDS [47] is a depression screening measure that is routinely used in home visiting. One MIECHV agency was unable to recover 1 mother’s baseline EPDS but did have this mother’s baseline total EPDS score. For this reason, the mother’s baseline EPDS could not be included in the baseline EPDS reliability analysis. The EPDS demonstrated good reliability at baseline (Cronbach alpha=.76) and follow-up (Cronbach alpha=.89) in this study. EPDS scores of mothers who received the video-delivered family therapy intervention were compared with those of depressed mothers who were previously enrolled in home visiting but refused treatment.

### Intervention

The video-delivered family therapy intervention consisted of 10, 30-min, weekly family therapy sessions that are concurrent with ongoing home visits [46]. The systemic treatment model was informed by DBT skills training for adolescents [55] and general systems theory [56]. It included skills that addressed 3 types of regulation: cognitive, emotion, and behavior [55]. The family therapy intervention included some skills from the following 4 DBT modules: mindfulness, distress tolerance, emotion regulation, and interpersonal effectiveness. The first author received written permission from the developers, Dr Jill Rathus and Dr Alec Miller, to adapt some skill teaching content from their model for use in the study intervention [55]. The study intervention addressed relational functioning but not parenting skills. Systemic interventions were woven into each skills module. The sessions were referred to as *skills training sessions* instead of *family therapy sessions* to reduce family stigma attitudes [46].

Home visitors were educated in how to use the HIPAA- compliant video-based communication technology (Vidyo) [57], and they educated families in its use [46]. Home visitors delivered most of the written skills handouts to families, but there were a few instances where these handouts had to be emailed to families because the families either misplaced the handouts or the home visitor was unable to deliver the handouts before the family therapy session.

### Statistical Analysis

#### Sample Size

A total of 13 families participated in the video-delivered family therapy intervention. The target sample size for the intervention group was 12 families, and it was determined in consultation with our external community stakeholders [46]. They recommended that we implement the protocol in the 2 MIECHV agencies with a small number of families to obtain early evidence of feasibility and acceptability in preparation for larger studies of potential effectiveness and scalability [46]. The historical comparison group was made up of 13 depressed mothers who were previously enrolled in home visiting but refused treatment. Pilot studies are used to assess the feasibility of an intervention for a larger-scale study, and the use of pilot study effect size for sample size estimation can lead to type I and II errors [58]. For these reasons, this study was not powered.

#### Feasibility and Acceptability

For the first aim, univariate statistics (eg, means and proportions) were used to characterize family baseline characteristics and indicators of feasibility (quality of the therapeutic alliance and retention). For quality of the therapeutic alliance, average therapist scores and family scores were calculated for each subscale on the Working Alliance Inventories-Short Forms [50,51] after family therapy sessions 6 and 10. Spearman rank correlations (rho) were performed to assess associations between family scores and therapist scores after family therapy sessions 6 and 10. For retention, the total number of attended family therapy sessions was calculated for each family and overall.

For acceptability (family responsiveness), univariate statistics and qualitative data analytic methods [59] were used. For family completion of homework assignments, the percentage of completed assignments was calculated overall and for each of the 4 skills modules [46]. Per the study protocol [46], averages were calculated for multiple-choice items and responses to the open-ended questions were coded [59] on the Satisfaction Questionnaire. A thematic analytic approach [59] was used for the semistructured, open-ended questions on the Satisfaction Questionnaire. Codes were grouped into categories to create themes, and the emergent themes were identified and summarized [59]. For the question on the most helpful skills, numeric values were assigned to each theme, and themes were rank-ordered by the percentage of mothers and family members that reported the skill was helpful.

#### Maternal and Family Outcomes

For the second aim, basic sample statistics (eg, means and proportions) were used to summarize family measures at baseline, post intervention, and at follow-up. Wilcoxon signed-rank tests were used to assess changes in maternal and family outcomes from baseline to post intervention and from baseline to follow-up.

The historical comparison group included depressed mothers who were previously enrolled in home visiting at the 2 MIECHV agencies but refused treatment. The mothers in the historical comparison group were matched with mothers who received the video-delivered family therapy intervention on 3 key baseline variables: EPDS baseline score, maternal age, and number of biological children. As we were unable to find previously enrolled mothers with EPDS scores that were exactly the same as baseline EPDS scores for mothers who received the study intervention, we matched mothers based on the similarity of their EPDS scores. A total of 39% (5/13) of comparison group mothers had EPDS scores that were exactly the same as those of intervention group mothers. A total of 39% (5/13) of comparison group mothers had EPDS scores that were within 1 point of those of intervention group mothers. A total of 15% (2/13) of comparison group mothers had EPDS scores that were within 2 points of those of intervention group mothers. Only 1 comparison group mother’s EPDS score was 3 points higher than that of an intervention group mother. A Wilcoxon signed-rank test was used to assess changes in EPDS scores in the matched pairs over a 6-month period.

## Results

### Family Characteristics

The intervention group family baseline characteristics are presented in Table 2. Mothers ranged in age from 18 to 25 years. Mothers’ family members ranged in age from 19 to 49 years. Most families (88% [23/26] individual family members) were white, and 77% (10/13) of families were enrolled in home visiting for 5 or fewer months.

**Table 2:** Family baseline characteristics (N=13).

Baseline characteristics			Statistics
**Mothers**			
	Age (years), mean (SD)		22.23 (1.92)
	First time mothers (pregnant and postdelivery), n (%)		8 (61)
	Pregnant, n (%)		5 (39)
	Months postpartum, mean (SD)		4.81 (4.24)
	**Highest level of education, n (%)**		
		11th grade	1 (8)
		High school graduate	7 (54)
		Some college	4 (31)
		College graduate	1 (8)
	Enrolled in school or employed for pay, n (%)		4 (31)
	**Mother’s relationship with family member, n (%)**		
		Partner/spouse	7 (54)
		Biological family member or close friend	6 (46)
	Severity of depressive symptoms, BDI-II^a^, mean (SD)		25.62 (10.98)
	**Severity of depressive symptoms, BDI-II, n (%)**		
		Minimal	3 (23)
		Moderately severe	4 (31)
		Severe	6 (46)
**Family members**			
	Age in years, mean (SD)		30.15 (10.61)
	Male, n (%)		7 (54)
	**Highest level of education, n (%)**		
		11th grade	1 (8)
		High school graduate	6 (46)
		Some college	3 (23)
		College graduate	2 (15)
		Master’s degree	1 (8)

^a^Beck Depression Inventory-second edition.

**Table 3:** Comparison of maternal baseline characteristics by study group.

Mothers (N=26)		Intervention group (n=13)	Historical comparison group (n=13)	*P* value
Age in years, mean (SD)		22.23 (1.92)	21.69 (1.55)	.44
**Race, n (%)**				.32
	White	12 (92)	0 (100)	—^a^
	Asian	1 (8)	0 (0)	—
Biological children, mean (SD)		1.00 (0.58)	1.23 (0.44)	.26
**Highest level of education, n (%)**				.17
	High school	8 (61)	12 (92)	—
	Some college	4 (31)	1 (8)	—
	College graduate	1 (8)	0 (0)	—
Serious relationship or married, n (%)		9 (69)	0 (100)	.10

^a^Not applicable.

There were no statistically significant differences in baseline characteristics of mothers who received the video-delivered family therapy intervention and those in the historical comparison group. Table 3 shows the baseline characteristics of mothers in both study groups.

The therapist consistently used a computer in her office to deliver the family therapy sessions using the HIPAA-compliant video-based communication technology. Families varied in the types of devices they used to participate in the family therapy sessions. About 31% (4/13) of families used only a tablet (eg, iPad and Amazon Fire) to participate in the family therapy sessions, and 31% (4/13) of families used only a computer to participate in these sessions. About 15% (2/13) of families used more than 1 device (eg, cell phone for some sessions and tablet for some sessions). Approximately 15% (2/13) of families used only a cell phone to participate in the family therapy sessions, and 1 family used an iPod Touch to participate in the sessions. Only 2 families had to reschedule family therapy sessions because of disruptions in internet service.

### Feasibility and Acceptability

For quality of the therapeutic alliance, family ratings on the 5-point subscales for the Working Alliance Inventories-Short Forms, goals (mean=4.8, SD=0.2), bond (mean=4.9, SD=0.3), and task (mean=4.4, SD=0.3), were very strong. As shown in Table 4, the family ratings were positively correlated with therapist ratings (*P*<.05) post intervention.

All families were retained, and no families dropped out of the intervention or the study. All mothers attended all 10 therapy sessions. Family members attended an average of 8.4 therapy sessions. One family included a home visited mother, her boyfriend, and her friend (also a home visited mother). After the fourth session, the couple decided to complete the family therapy without the other home visited mother. For this reason, the other home visited mother attended the remaining 6 therapy sessions without a family member.

All mothers and family members completed homework assignments from each of the 4 skills modules. Only 1 mother reported that she would prefer in-person delivered sessions, instead of video-delivered sessions, in the future. For the qualitative item on the helpfulness of the skills on the Satisfaction Questionnaire, all families reported that the mindfulness skills were most helpful for them, followed by emotion regulation (9/13, 69%), distress tolerance (6/13, 46%), and interpersonal effectiveness (5/13, 39%). Families did not report any suggestions for changes to the intervention. No families reported any complaints. The results for the remaining quantitative items on the Satisfaction Questionnaire are included in Table 5.

### Maternal and Family Outcomes

**Figure 1:**
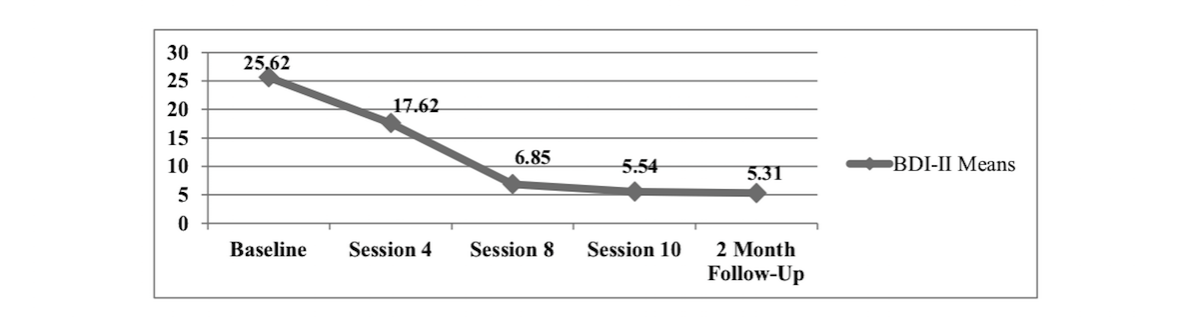
Decrease in maternal depressive symptoms by time point (n=13). BDI-II: Beck Depression Inventory-second edition.

The results showed statistically significant reductions in maternal depressive symptoms (*P*=.001) at the 2-month follow-up. Figure 1 shows the decrease in maternal depressive symptoms by time point.

The results showed statistically significant improvements in family functioning (*P*=.02) and cognitive reappraisal (*P*=.004) at the 2-month follow-up. Table 6 includes the impacts of the video-delivered family therapy intervention on maternal and family outcomes.

Although maternal school enrollment and job attainment were not proposed outcomes in this study, 39% (5/13) of mothers either enrolled in school or were employed for pay by the 2-month follow-up. Furthermore, 31% (4/13) of mothers who were in school or employed for pay at baseline maintained this status post intervention. This finding indicates that the significant reduction in maternal depressive symptoms resulted in improved functioning.

This study included a historical comparison group of 13 mothers who were previously enrolled in home visiting and screened positive for depression but refused treatment. Home visiting programs routinely use the EPDS to screen for maternal depressive symptoms at different time points. The EPDS scores of mothers who received the video-delivered family therapy intervention were compared with those of previously enrolled depressed mothers who refused treatment. Table 7 includes the differences in EPDS scores by study group.

**Table 4:** Correlations between therapist and family therapeutic alliance ratings post intervention.

Working Alliance Inventories-Short Forms Subscale	Correlation coefficient	*P* value
Goals	.571	.04
Bond	.607	.03
Task	.567	.04

**Table 5:** Family satisfaction with video-delivered family therapy intervention (N=13).

Questionnaire item	Strongly agree, n (%)	Agree, n (%)
I am satisfied with the video-delivered skills training that I received in this study	13 (100)	0 (0)
In the sessions, I learned skills to help me stay focused on the present moment	13 (100)	0 (0)
In the sessions, I learned skills to help me to manage problems that could not be immediately resolved	10 (77)	3 (23)
In the sessions, I learned skills to help me to manage my emotions	12 (92)	1 (8)
In the sessions, I learned skills that strengthened my relationships	12 (92)	1 (8)
The video-based technology was convenient and easy to use	11 (85)	2 (15)

**Table 6:** Video-delivered family therapy intervention impacts on outcomes.

Families (N=13)		Baseline, mean (SD)	Post intervention, mean (SD)	Follow-up, mean (SD)	*P* value
Maternal depressive symptoms^a^		25.62 (10.98)	5.54 (4.68)	5.31 (4.35)	≤.001
Family functioning^b^		4.82 (1.05)	5.96 (0.72)	5.65 (0.85)	.02
**Family emotion regulation**					
	Cognitive reappraisal^c^	4.47 (0.76)	5.66 (0.88)	5.49 (0.40)	.004
	Expressive suppression^c^	3.47 (0.85)	3.51 (1.03)	3.56 (0.84)	.65

^a^Beck Depression Inventory-second edition.

^b^Protective Factors Survey-Family Functioning/Resiliency subscale item mean.

^c^Emotion Regulation Questionnaire subscale total item means.

**Table 7:** Comparison of maternal depressive symptoms by study group.

Depressive symptoms^a^	Intervention group, mean (SD)	Historical comparison group, mean (SD)	*P* value
Baseline	13.62 (4.75)	13.46 (4.33)	.57
Follow-up^b^	6.08 (6.22)	15.31 (5.01)	≤.001

^a^Depressive symptoms were measured by home visitors using the Edinburgh Postnatal Depression Scale.

^b^The follow-up period was about 6 months.

## Discussion

### Principal Findings

This study explored the feasibility, acceptability, and preliminary impacts of a video-delivered family therapy intervention for home visited young mothers with perinatal depressive symptoms and their adult family members. Our findings showed that mothers experienced a statistically significant reduction in depressive symptoms, and families experienced statistically significant improvements in family functioning and the emotion regulation strategy of cognitive reappraisal. This study demonstrated the preliminary safety of the video-delivered family therapy for home visited depressed young mothers as our results showed that maternal depressive symptoms consistently decreased from baseline to the 2-month follow-up (see Figure 1). We found that the significant reduction in maternal depressive symptoms also resulted in an improvement in maternal occupational functioning in that 39% (5/13) of mothers either enrolled in school or were employed for pay by the 2-month follow-up. Furthermore, 31% (4/13) of mothers who were either enrolled in school or employed for pay at baseline maintained this status at the 2-month follow-up.

Some technology-based interventions (eg, short message service text messages, Web-based peer support, and internet therapy) have been tested in perinatal and postpartum women with depression and have been shown to be effective in reducing their depressive symptoms [60-63]. For example, a recent systematic review and meta-analysis showed that therapist- supported internet-based cognitive behavioral therapy significantly reduced depressive symptoms in postpartum women [64]. The video-delivered family therapy intervention presented in this study is unique, and to our knowledge, this is the first study of a technology-based family therapy intervention with home visited women with perinatal depressive symptoms.

To date, 4 individual-level psychosocial interventions have been developed for and tested in home visited depressed mothers [21-24]. Either mothers travel to receive the intervention [23] or providers travel to mothers’ homes to deliver these interventions [21,22,24]. Young mothers experience family conflict [25-31,33-35], and these interventions do not include family members to resolve the conflict. Although maternal depressive symptoms are primary outcomes in these interventions [21-24], systemic interventions are not included to create second-order change by shifting family dynamics to improve family functioning. Furthermore, the video-delivered family therapy intervention differs from other similar family therapy models used with home visited families [65-67] in that it does not focus on parenting practices or parenting skills and it is delivered using HIPAA-compliant video-based communication technology. This study fills an important gap in the existing literature in that it provides preliminary support for a technology-based family therapy intervention that targets home visited young mothers with clinically significant perinatal depressive symptoms.

This study demonstrated the feasibility and acceptability of the video-delivered family therapy intervention. We found support for all 3 of our hypotheses. First, we hypothesized that mothers would show a clinically meaningful reduction in depressive symptoms at the 2-month follow-up [46]. The results showed that mothers demonstrated a statistically significant reduction in depressive symptoms at the 2-month follow-up. Second, we hypothesized that mothers who received the video-delivered family therapy intervention would show a greater reduction in depressive symptoms when compared with depressed mothers who were previously enrolled in home visiting but refused treatment [46]. We found that mothers who received the video-delivered family therapy intervention had a significantly greater reduction in depressive symptoms than did those who received home visiting but refused treatment for depression.

Finally, we hypothesized that families would show clinically meaningful improvements in family functioning and emotion regulation at the 2-month follow-up [46]. Our results showed that families demonstrated statistically significant improvements in family functioning and 1 emotion regulation strategy, cognitive reappraisal. Families did not show a statistically significant improvement in expressive suppression. We believe that there are 2 possible reasons why they did not show a significant improvement in expressive suppression. First, the Emotion Regulation Questionnaire-Expressive Suppression subscale did not demonstrate very strong reliability in our sample of families (Cronbach alpha=.68) at baseline. Second, their scores were similar to the norm [54] for this subscale.

The significant reduction in maternal depressive symptoms and significant improvements in family functioning and cognitive reappraisal hold promise for future research on this intervention. Concordantly, we plan to conduct a randomized trial of the study intervention to formally test its impacts on these outcomes.

### Limitations

This study had several limitations. First, the results must be interpreted with caution, given the small sample size. Second, we were unable to conduct a long-term follow-up because of the short-term nature of the grant received to conduct this study. Third, this study did not include a control group. Fourth, the majority of the participants were white, and the lack of ethnic diversity limits the generalizability of our findings. Fifth, most of the participants graduated from high school, and our findings may not generalize to those with lower levels of education. Sixth, the study included heterosexual mothers, and although we did not exclude mothers who identify as lesbian from participating in the study, it is possible that the findings may not be generalizable to mothers who identify as lesbian. Finally, the findings from this type of study intervention may not be generalizable to home visited families in urban communities, given they may face fewer logistical barriers and may prefer traveling to providers’ offices for family therapy.

### Conclusions

There is a scarcity of research on the use of HIPAA-compliant video-based communication technology to deliver family therapy for treatment of perinatal depression in rural regions of the United States. The studies that have been conducted on this method of delivery focus on family therapy interventions for behavioral problems in children [41-44], but none have targeted perinatal depressive symptoms in young mothers with family discord [45]. The video-delivered family therapy intervention presented in this study is unique in that it addresses individual-level and family-level cognitive, emotion, and behavior regulation. Although the results of this study are very promising, a large randomized controlled clinical trial with a longer follow-up period is needed to confirm the effectiveness of this novel family therapy intervention. We plan to conduct future research studies on the effectiveness of the video- delivered family therapy intervention with mothers with only perinatal depressive symptoms and those with perinatal depressive symptoms and other comorbid conditions (eg, anxiety and substance abuse).

## Abbreviations

BDI-II: Beck Depression Inventory-second edition
CAGE: cut down-annoyed-guilty-eye opener
DBT: dialectical behavior therapy
EPDS: Edinburgh Postnatal Depression Scale
HIPAA: Health Insurance Portability and Accountability Act
IRB: institutional review board
MIECHV: Maternal, Infant, and Early Childhood Home Visiting
PI: principal investigator
