# Addendum to the Acknowledgements: Video-Delivered Family Therapy for Home Visited Young Mothers With Perinatal Depressive Symptoms: Quasi-Experimental Implementation-Effectiveness Hybrid Trial

**DOI:** 10.2196/13636

**Published:** 2019-03-07

**Authors:** Fallon Cluxton-Keller, Melony Williams, Jennifer Buteau, Craig L Donnelly, Patricia Stolte, Maggie Monroe-Cassel, Martha L Bruce

**Affiliations:** 1 Department of Psychiatry Geisel School of Medicine at Dartmouth College Lebanon, NH United States; 2 FRC Claremont, NH United States; 3 FRC Gorham, NH United States

The authors of “Video-Delivered Family Therapy for Home Visited Young Mothers With Perinatal Depressive Symptoms: Quasi-Experimental Implementation-Effectiveness Hybrid Trial” (JMIR Ment Health 2018;5(4):e11513) wish to add the following funding statement to their Acknowledgments section:

Research reported in this publication was supported by The Dartmouth Clinical and Translational Science Institute, under award number UL1TR001086 from the National Center for Advancing Translational Sciences (NCATS) of the National Institutes of Health (NIH). The content is solely the responsibility of the authors and does not necessarily represent the official views of the NIH.

The correction will appear in the online version of the paper on the JMIR website on March 7, 2019, together with the publication of this correction notice. Because this was made after submission to PubMed, PubMed Central, and other full-text repositories, the corrected article also has been resubmitted to those repositories.

