# Reference Correction: Video-Delivered Family Therapy for Home Visited Young Mothers With Perinatal Depressive Symptoms: Quasi-Experimental Implementation-Effectiveness Hybrid Trial

**DOI:** 10.2196/15661

**Published:** 2019-09-18

**Authors:** Fallon Cluxton-Keller, Melony Williams, Jennifer Buteau, Craig L Donnelly, Patricia Stolte, Maggie Monroe-Cassel, Martha L Bruce

**Affiliations:** 1 Department of Psychiatry Geisel School of Medicine at Dartmouth College Lebanon, NH United States; 2 FRC Claremont, NH United States; 3 FRC Gorham, NH United States

In “Video-Delivered Family Therapy for Home Visited Young Mothers With Perinatal Depressive Symptoms: Quasi-Experimental Implementation-Effectiveness Hybrid Trial” (JMIR Ment Health 2018;5(4):e11513) by Cluxton-Keller et al, references 4 and 18 were duplicates.

The original reference 4 was as follows:

Duggan AK, Berlin LJ, Cassidy J, Burrell L, Tandon SD. Examining maternal depression and attachment insecurity as moderators of the impacts of home visiting for at-risk mothers and infants. J Consult Clin Psychol 2009 Aug;77(4):788-799.

It has been replaced by the following new reference:

Caldera D, Burrell L, Rodriguez K, Crowne SS, Rohde C, Duggan A. Impact of a statewide home visiting program on parenting and on child health and development. Child Abuse Negl 2007 Aug;31(8):829-52.

The correction will appear in the online version of the paper on the JMIR website on September 18, 2019, together with the publication of this correction notice. Because this was made after submission to PubMed, PubMed Central, and other full-text repositories, the corrected article also has been resubmitted to those repositories.
